# Miscellaneous Surgical Essays

**Published:** 1781-05

**Authors:** 


III. *Mifcellaneous Surgical Effays, by* J. L. Schmucker. *(Continued from page* 246.*)*

XV. IN this paper feveral cafes are related by Mr. Schumacher. The firft is a cafe of fiftula in ano, that extended an inch within the rectum. After performing the operation ufual in fuch cafes, our author difcovered an opening, that afforded a paſſage to the urine. He introduced a probe into this channel, and dilated it as far as the bladder. A ſmart fever ſucceeded this operation, and laſted ten days, during which time not a drop of urine was diſcharged through the urethra. Another fiſtula of nine inches in length was diſcovered running towards the buttock, and for this alſo the uſual operation was had recourſe to. After the fever and inflammation had ſubſided, the urine began again to take its natural courſe through the urethra, and at length it entirely ceaſed to flow through the wounds.

In another patient, Mr. Schumacher extracted a polypus from the noſe, that weighed three ounces and a half, and extended a conſiderable way into the fauces. The profuſe hæmorrhage occaſioned by the removal of this maſs, was ſtopped by injecting the following liquor: ℞. Bol. armen. pp. ℥j. ſucc. catuchu. terræ ſigillat. āā ℥ſs. limat. clavellator. ʒvj. M. affunde ſpir. vin. rectificatiſſim. ℔j. digere per tres dies et cola.

Mr. Schumacher ſpeaks of a third caſe, in which the ſkin of the ſcrotum and penis, after having been deſtroyed by a gangrene of thoſe parts, was completely renewed.

The author next mentions the hiſtory of a patient, who, after having been for ſeveral years much troubled with the hæmorrhoids, became melancholic, and occaſionally ſubject to *deliquium animi.* He had a conſtant trembling of his right leg, and at length ſymptoms of apoplexy, which ended in death. On diſſection, a ſteatomatous tumour was found between the dura and pia mater, that weighed three ounces.

The laſt caſe related by Mr. Schumacher, is that of a patient whoſe complaints originated from a wound with a broad ſword, that penetrated through the os bregmatis to the brain. The wound healed, but left the patient ſubject to vertigo and head-ache. During ſixteen years, however, he enjoyed a tolerable ſtate of health, relieving his head-ache by occaſional venæſections. At the end of that time he began to be ſubject to apoplexy, which at length terminated fatally. On diſſection, it was diſcovered that the wound of the parietal bone was not cloſed as had been ſuppoſed, but was open through its whole length. The cortical ſubſtance of the brain was of a gelatinous texture; the medullary ſubſtance hard, and abounding with large blood veſſels; the ventricles were filled with coagulated blood and ſerum, and the left ventricle tricle contained a ſteatomatous tumour of the ſize of a nutmeg.

XVI. *A Caſe of Hemiplegia, occaſioned by Blood extravaſated within the Cranium, by* Mr. Wurm. We have here the caſe of a ſoldier, who often loſt his underſtanding for a ſhort ſpace of time. Previous to the paroxyſm, he conſtantly complained of a particular ſenſation over the margin of the left orbit. It was at length diſcovered, that about three weeks previous to theſe attacks, he had received a violent contuſion over his left eye; and on examining the part, our author obſerved the appearance of a cicatrix. An inciſion was made through it; and the night following the left ſide of the patient's body became paralytic. The trepan was then had recourſe to; a conſiderable coagulum of freſh extravaſated blood was found within the cranium; and ſoon after its removal the ſymptoms diſappeared.

XVII. *A Caſe of a Wound of the Head, by* Mr. Geiſler. A ſoldier who attempted to ſhoot himſelf, put the orifice of his firelock ſo near to his forehead, that the exploſion affected chiefly the integuments, and made but a ſlight impreſſion on the bones of the head. In the courſe of a few days, an oedematous ſwelling

ſpread over the whole head, the ſkin ſeparated from both the parietal bones, and a conſiderable ſuppuration took place. Openings were made in different parts, in order to give a free vent to the matter, and as the ſuppuration diminiſhed, the ſkin was preſſed to the bones by compreſſes, and ſoon united with them again, ſo that only a ſmall portion of bone, of about the ſize of a ſhilling, exfoliated.

XVIII. *Of a Concuſſion of the Brain, by* Mr. Köhler.—The cure in this caſe was effected chiefly by repeated bleedings, but ſlowly. It was remarkable that the patient found himſelf worſe as often as warm fomentations were applied to his head. The editor of the work, Mr. Schumacher, takes occaſion from this circumſtance to point out the bad effects of warm, and the utility of cold applications in wounds of the head.

XIX. *Of a fatal wound of the forehead, by* Mr. Piſtor. The patient, whoſe caſe is here related, was wounded in the forehead with a knife that penetrated the brain. He would not ſubmit to the application of the trepan, and a conſiderable ſuppuration of the brain took place, notwithſtanding which the patient lived ſeventy-ſix days after the accident.

XX. *Of a gun ſhot wound through the ſinus frontalis, by* Mr. Ramdohr.—In this caſe the ball paſſed into the brain. Ths patient fell down at the time he received the wound, but ſoon grew better, and during the ſpace of four months remained ſo well that no perſon would have doubted of his perfect recovery, if it had had not been known that the ball was ſtill within the cranium. At the end of that time he became lethargic, affected with convulſions, and died. After his death the ball was found in the middle of the medullary ſubſtance, half an inch above the left anterior ventricle.

XXI. *The caſe of a young man whoſe hand was torn off at the joint of the wriſt by the wheel of a mill, by* Mr. Rieſenbach.—In this caſe the ends of the bones of the fore arm were laid bare for upwards of an inch, and two conſiderable excavations were obſerved in the fleſhy parts, occaſioned by the loſs of muſcles that had been carried away with the hand. Nature ſoon threw off the prominent part of the ulna, but the ſeparation of the radius proceeding more ſlowly, our author judged it adviſeable to ſaw off the extremity of that bone. This was accordingly done, and the wound healed ſoon after.

XXII. *A luxation of the vertebræ colli succesfully treated, by* Mr. Sellie.—In this case the patient fell backwards from his horse and remained senseless on the ground. His head was so extremely moveable, that it required the assistance of a person to confine it. His face was much swelled, his mouth open, and his respiration so slow that he could hardly be said to breathe more than once in a minute, his pulse at the same time being scarcely perceptible. The vertebræ being evidently luxated backwards, the author directed one of his assistants to take hold of the patient's head, while another did the same with his shoulders and lower part of his neck. Both made an extension at the same time; and increased it by degrees; when it was sufficient Mr. Sellie pressed the luxated vertebræ inwards into their right place. The head immediately acquired more firmness; the volatile spirit was rubbed into his neck, and a dose of the tincture thebaica administered immediately after the operation. In a few minutes the patients breathing became freer, the pulse stronger, and all the symptoms gradually disappeared, so that on the eighth day after the accident he was perfectly recovered.

XXIII. *Of a luxation of the last vertebra dorsi and first vertebra lumborum, by* Mr. Riediger.— The accident here spoken of was occasioned by a considerable weight that fell upon the patient's back. The last vertebra of the back was luxated outward, and on the right side, three fingers breadth from the first lumbar vertebra. In order to reduce it, the patient was placed in a straight line, upon his belly, in a bed, and when the extension was sufficient, the bone by suitable pressure, though not without much difficulty, was brought back into its natural position; but no sooner was the extension discontinued than it resumed its former situation. This was prevented from happening again by a constant pressure with the hand for some time, and afterwards by means of compresses, and bandage. The patient continued in this extended posture on his belly for twenty days, after which he was allowed to lie upon his back, and in six weeks the cure was complete.

XXIV. *Of a violent colic occasioned by a sharp piece of bone in the intestines, by* Mr. Sponitzer.— The pain in this case was extremely severe, and attended with fever and vomiting. The inflammatory symptoms were lessened by repeated venæsections, and by means of purgative remedies the the ſeat of the pain was gradually removed lower down till at length it reached the rectum, and the patient then voided blood with his fæces. This induced our author to introduce his finger into the rectum, and by this means he diſcovered a hard ſubſtance which was with difficulty extracted, and proved to be a ſharp-pointed piece of bone, two inches in length.

XXV. *Of a gun ſhot wound through the lungs, ſucceſsfully treated, by* the ſame.—In this patient the ball paſſed through both lobes of the lungs, and fractured the ſcapula and one of the ribs, The ſplinters of bone were carefully extracted, and in ten weeks time by means of repeated blood-letting, the patient, notwithſtanding his intemperate uſe of brandy, recovered. He is now alive and well, though the accident happened twenty years ago. It is remarkable that the anterior wound where a part of the rib an inch long was loſt, formed a cicatrix which is moved at each inſpiration.

XXVI. *Of an abſceſs of the abdomen, by* the ſame.—In this caſe a painful tumour aroſe on the right ſide of the belly, and at length ſuppurated and produced a fiſtulous ulcer. At firſt fæces paſſed through it, and afterwards indigeſted aliment, ſo that the diſcharge by the anus was entirely entirely ſtopped. After a while, however, compreſſion on the fiſtula diſpoſed it to heal, and the fæces began to reſume their natural courſe again without any other remedy.

XXVII. *Of a ſingular diſeaſe of the Elbow joint*, *by* Mr. Gödücke.—The complaint here ſpoken of was brought on by a ſlight contuſion. The joint ſwelled, became painful, and was ſuppoſed to contain matter. On cutting into this tumour our author, inſtead of pus, diſcovered a ſoft excreſcence, which he extracted without any conſiderable hæmorrhage, but it repeatedly grew again and was as often extirpated, till at length it became cancerous, and the patient died.

XXVIII. *Caſe of a Fiſſure of the Tibia*, *by* the ſame.—This accident was occaſioned by the kick of a horſe. At firſt no injury was obſervable in the bone, but the patient could not make uſe of his leg; at length a hollow line was diſcovered, which diſappeared in ſix weeks, and the patient recovered the uſe of his limb.

XXIX. *Of an Abſceſs of the Back, occaſioned by an Inflammation of the Lungs*, *by* the ſame.—In this caſe, the patient who ſome time before had been troubled with an inflammation of the lungs, complained of violent pain in his right ſide, near the ſpine, and at about the ſixth or ſeventh true ribs rib. As the danger feemed urging, our author, notwithftanding there was no fwelling externally, ventured to make an incifion at this part. A confiderable quantity of fœtid pus was difcharged through this opening, and in the courfe of a few weeks the patient found himfelf well.

XXX. *An Account of a fatal Suppuration of the Mufcles of the Calf of the Leg, by* Mr. Kühn. —A man, after leaping over a ditch, was feized with an acute pain in the calf of his leg, which continued a year and a half, when a fwelling appeared externally, which increafed in a fhort time fo much that it feemed likely to burft. The patient for fome time refufed to have it opened; at length, however, he confented; but it was too late, as the mufcles were deftroyed, and the bone was in a carious ftate. The night fucceeding the operation, the patient was attacked with fever and delirium; the next day a mortification came on, and on the fifth the patient died.

XXXI. *Of an inflammatory Sore Throat, fucceeded by Abfceffes in different Parts of the Body, by* the fame.—Mr. Kühn relates a cafe of inflammatory fore throat that ended in fuppuration. Two pounds of pus were difcharged from the abfcefs; and a few weeks afterwards two other abfceffes formed, one on the patient's arm, and the other on his fore arm. The former yielded eleven, and the latter fifteen ounces of pus. Thefe were fucceeded by abfceffes in the foot, thigh, and leg, each of which afforded a confiderable difcharge of matter, particularly the latter; three pounds eleven ounces of pus having been evacuated from it. After thefe fucceffive fuppurations, the patient recovered by means of the Peruvian bark combined with the *radix ferpentaria*, of which we are told he took thirty-three drachms daily.

XXXII. *An Account of a dangerous Wound of a Child's Hand fuccefsfully treated, by* Mr. Jung. —In this cafe all the bones and mufcles were cut through, and the hand was fufpended only by part of the integuments, and by the tendon of the flexor indicis. The child was attacked with the fmall-pox foon after the accident, but the cure was completed in little more than two months without any exfoliation; all the fingers, however, remained ftiff.—Mr. Hoffmann, we are told, was equally fuccefsful in a fimilar wound of the fore arm. In this cafe the bones, and moft of the mufcles were divided, but the large veffels remained untouched. The cure was per-

 formed formed in ten weeks without exfoliation, but the arm remained a little crooked.

XXXIII. *Of a large Furunculus on the Back, by* Mr. Schopper.—The *furunculus* here ſpoken of was as large as a plate, and a great part of it was in a gangrenous ſtate. The patient had a violent fever with delirium. Mr. Schopper ſcarified not only the mortified part, but likewiſe the ſound edges of the furunculus, and ſprinkled it with ſal ammoniacum and Peruvian bark, giving at the ſame time the latter internally. By theſe means a ſeparation was produced, and the patient recovered.

Mr. Schmucker, the editor of this volume, has added ſome very intereſting remarks on this ſpecies of *furunculus*, which he obſerves may be called *furunculus gangrænoſus*. At its firſt appearance it is not larger than a cheſnut, after which it ſpreads daily till it is as large as a plate, but without becoming ſoft. Through its whole courſe it is accompanied with a violent inflammatory fever, and ſometimes with delirium. No topical applications will bring it to ſuppuration. It continues uniformly hard, inflamed, and painful, and if opened yields only an ichorous diſcharge. Its moſt uſual ſeat is in thoſe parts of the ſkin where the adipoſe membrane is thickeſt, as as the neck, back, hypochondriac and iliac regions; of all these our author considers the neck as the most dangerous part it can occur in. He ascribes the cause of this *furunculus* to a gouty matter, having never observed this disease in any but gouty people. In general the furunculus breaks of itself by several small openings, through which exsudes a yellowish and green ichor. Mr. Schmuecker has seen thirty such openings. He advises us to enlarge them without delay, otherwise the ichor will corrode all the surrounding parts to the bone, the inflammatory will change into a putrid fever, and death will be unavoidable. The cure we are told, depends on early venæsection, which it will sometimes be necessary to repeat five or six times during the first three days, in order to moderate the inflammatory symptoms. When a gangrene has taken place, we are directed to obviate it by early and sufficient incisions.

XXXIV. *An Account of the ill Effects of fresh baked Bread, by* Mr. Horn.—This paper contains the cases of two soldiers who died soon after eating immoderately of fresh baked bread. They complained of great uneasiness at the stomach, and puked frequently, but without evacuating any bread; their bellies became extremely hard and and tumid, and their pulſe hardly perceptible. On examining their bodies after death, the inteſtines were found extremely diſtended with air, and ſingularly contorted.

As cattle are occaſionally ſubject to a ſimilar inflation, and the common practice in ſuch caſes is to evacuate the air by plunging a trochar into the abdomen, our author aſks, whether, under ſimilar circumſtances, ſuch a mode of relief would be adviſeable in the human ſubject?

XXXV. *Of the Cure of a ſpurious Aneuriſm, by* Mr. Cramer.—The aneuriſm here ſpoken of was as large as a hen's egg, and the cure was effected in about four months by compreſſion.

XXXVI. *An Account of a Man who ſwallowed Pins, Nails, &c. by* Mr. Block.—This man ſwallowed 102 pins, 150 nails, ſeveral hair-pins, pieces of glaſs, buckles, money, &c. all which paſſed off ſucceſſively by ſtool, without producing any other inconvenience than frequent puking.

XXXVII. *Of a Caries of the Scull, by* Mr. Sellie.—A wound on the upper part of the os frontis was completely cured in about a fortnight; but ſome time afterwards the patient complained of a violent pain in that part, and at length a ſwelling took place over the whole head, and was ſucceeded fucceeded by hectic fever, locked jaw, and a fuppuration. On opening the abfcefs a great part of the cranium was found to be carious. As the difeafe was fufpected to be venereal, mercurial frictions were prefcribed, and in about three months the patient was cured.

XXXVIII. *Of a Mortification of the Vagina after a difficult Labour, by* Mr. Hagen.—In this cafe the urethra was likewife deftroyed, notwithftanding which the patient recovered.

XXXIX. *Of a Prolapfus of the Colon, by* the fame.—The colon in this cafe defcended through the anus, and being neglected, mortified and proved fatal to the patient.

XL. *An Account of a fpitting of Blood in an Infant, by* Mr. Riefenbach.—The patient was only five days old, and a coffee-cupful of blood was difcharged from the mouth without any bad confequence.

XLI. *Of a difficult Labour, occafioned by a Prolapfus of the Vagina, by* Mr. Giefeman.—In this cafe the opening in the prolapfed vagina was not larger than a fhilling, and at each labour pain the whole uterus with the vagina and child feemed, as our author expreffes himfelf, to fall out. The woman from her exhaufted ftate feeming to require fpeedy relief, Mr. Giefeman introduced a hollow probe probe into the opening of the vagina, which he cut throughout its whole length, by which means the delivery was eafily accomplifhed, and the woman, we are told, has fince borne three children.

XLII. *Of a Suppuration of the Omentum, by* Mr. Dibel.—A woman far advanced in her pregnancy received a violent blow on the navel, which occafioned very fevere pain. The pain was relieved by venæfection, and about three weeks after the accident the patient was fafely delivered; but the labour renewed the pain at the navel again, and it was not long before an abfcefs began to appear in the left groin, which fuppurated and difcharged a confiderable quantity of pus and blood mixed with pieces of corrupted omentum. After this the patient foon recovered.

