# Errata

**Published:** 1781-06

**Authors:** 


					-p. 31,4, 1. 16, for Mr. Schumicher read Mr. J>chmncker.-

				

